# The effect of growth hormone on the metabolome of follicular fluid in patients with diminished ovarian reserve

**DOI:** 10.1186/s12958-023-01073-x

**Published:** 2023-02-27

**Authors:** Fan He, Fang Wang, Yang Yang, Zhi Yuan, Chengguang Sun, Heng Zou, Huijia Chen, Hongliang Yi, Shan Hu Gao, Shen Zhang, Lina Hu, Ting-li Han

**Affiliations:** 1 The Center for Reproductive Medicine, Obstetrics and Gynecology Department, The Second Affiliated Hospital of Chongqing Medical University, Chongqing, 400010 China; 2 Joint International Research Lab for Reproduction and Development, Ministry of Education, Chongqing Medical University, Chongqing, 400010 China; 3 Reproduction and Stem Cell Therapy Research Center of Chongqing, Chongqing, 400010 China; 4 Department of Reproduction and Infertility, Chengdu Women’s and Children’s Central Hospital, School of Medicine, University of Electronic Science and Technology of China, Chengdu, 611731 China; 5 Obstetrics Department, The First Affiliated Hospital of Chongqing Medical University, Chongqing, 400042 China; 6 Mass Spectrometry Centre of Maternal-Fetal Medicine, Chongqing Medical University, Chongqing, 400010 China

**Keywords:** Growth hormone, Diminished ovarian reserve, Follicular fluid, Metabolomics, Gas chromatograph-mass spectrometry

## Abstract

**Background:**

Increasing evidence supports that the co-treatment with growth hormone (GH) enhances ovarian response and oocyte quality during controlled ovarian stimulation (COS) in patients with diminished ovarian reserve (DOR). The composition of follicular fluid (FF) plays an essential role in oocyte development and mirrors the communication occurring between the oocyte and follicular microenvironment. However, the effect of GH on the FF metabolome remains unclear.

**Methods:**

This prospective observational study recruited DOR patients undergoing in vitro fertilization (IVF) cycles with minimal stimulation protocol for COS. Each patient receiving GH co-treatment was matched to a patient without GH co-treatment by propensity score matching. The FF was collected after isolating oocytes and assayed by gas chromatograph-mass spectrometry (GC-MS) metabolomics. The Pearson correlation was performed to evaluate the relationship between the number of oocytes retrieved and the levels of differential metabolites. The KEGG database was used to map differential metabolites onto various metabolic pathways.

**Results:**

One hundred thirty-four FF metabolites were identified by GC-MS metabolomics. Twenty-four metabolites, including glutathione, itaconic acid and S-adenosylmethionin (SAM) showed significant differences between the GH and control groups (*p*-value < 0.05 and *q*-value < 0.1). In addition, the number of oocytes retrieved was significantly higher in the GH group compared to the control group (3 vs 2, *p* = 0.04) and correlated with the levels of five differential metabolites. Among them, the levels of antioxidant metabolite itaconic acid were upregulated by GH administration, while SAM levels were downregulated.

**Conclusions:**

The co-treatment with GH during COS may improve oocyte development by altering FF metabolite profiles in DOR patients. However, given the downregulation of SAM, a regulator of genomic imprinting, the potential risk of imprinting disturbances should not be neglected.

**Supplementary Information:**

The online version contains supplementary material available at 10.1186/s12958-023-01073-x.

## Background

Diminished ovarian reserve (DOR), also called as poor ovarian reserve (POR), refers to reduced oocyte quantity or quality in women of reproductive age [1]. Women with DOR exhibit decreased response to ovarian stimulation or reduced fecundity compared to women of comparable age [1]. The prevalence of DOR is estimated at 10% among infertile women. Patients with DOR usually seek assisted reproductive technology as fertility treatment. Unfortunately, DOR is associated with ovarian hypo-response, a higher chance of cycle cancellation, reduced numbers of oocytes retrieved and embryos, lower rates of embryo implantation, clinical pregnancy and live birth, and higher rates of miscarriage and aneuploid in in vitro fertilization (IVF) cycles [2–4]. Various adjuvant therapies, including growth hormone (GH), testosterone, dehydroepiandrosterone (DHEA), L-arginine, and coenzyme Q10, have been developed to improve IVF outcomes in DOR patients [5–9].

GH, a single-chain peptide secreted by somatotrophic cells within the anterior pituitary in a pulsatile pattern, is involved in regulating protein synthesis, cell proliferation and metabolism [10]. The expression of GH and its receptor (GHR) was detected in human oocytes, granulosa and stromal cells, implying that GH may play a critical role in human reproduction [11]. Thirty years ago, GH was first prescribed to improve the ovarian response in patients who had previously responded sub-optimally to standard ovarian stimulation regimens for IVF [12]. Several recent studies showed that GH treatment improved IVF outcomes of patients with DOR or poor ovarian response, including clinical pregnancy and live birth, probably through the beneficial effect on the number of oocytes retrieved and good-quality embryo formation [13–15]. The beneficial role of GH treatment in DOR patients undergoing IVF was further supported by two systematic reviews including randomized controlled trials [16, 17]. GH was demonstrated to improve ovarian response or oocyte quality through upregulating the density of GHR in human oocytes and granulosa cells [18, 19], stimulating the secretion of insulin like growth factor 1 [20], and promoting follicle-stimulating hormone (FSH)-mediated ovarian steroidogenesis [21]. However, the underlying mechanisms have not been fully elucidated.

Follicular fluid (FF) is deemed as a transudate of serum components and the secretions of theca and granulosa cells in ovarian follicles [22], constituted by a variety of molecules, including proteins, steroid hormones, metabolites, and polysaccharides. FF surrounding the oocyte forms an essential element for the follicular microenvironment and plays a crucial role in oocyte development [23]. Links have been established between FF metabolites and the clinical outcomes of patients undergoing IVF [24–26]. Notably, FF metabolite profiles of DOR patients are significantly different from that of women with a normal ovarian reserve [27, 28]. These suggested that GH may improve IVF outcomes of DOR patients by changing FF metabolite profiles.

This study aimed to investigate the effect of GH adjuvant treatment on the FF metabolome of DOR patients undergoing IVF and analyze the potential metabolic pathways in which the differential metabolites are involved.

## Materials and methods

### Study design and participants

This prospective observational study was conducted in the Center for Reproductive Medicine at the Second Affiliated Hospital of Chongqing Medical University. The consecutive IVF cycles with minimal stimulation protocol for controlled ovarian stimulation (COS) between May 2019 and November 2019 were screened for patients with DOR, defined as serum anti-Müllerian hormone (AMH) level below 1.1 ng/ml or antral follicle count (AFC) below seven [29, 30]. We excluded those DOR patients with: 1) body mass index (BMI) ≥24 kg/m^2^; 2) a history of endometriosis, ovarian surgery, polycystic ovary syndrome, or other endocrine or autoimmune dysfunction; 3) other adjuvant treatment including DHEA and melatonin; 4) previous IVF attempts more than once; or 5) less than 3 months since last IVF attempt. The AFC and basal sex hormone levels were evaluated on days 2-4 of the menstrual cycle. The propensity score matching (PSM) was performed to reduce the potential bias from confounding variables including age and basal FSH levels. Each patient receiving GH co-treatment was matched to a patient without GH co-treatment by PSM. The patients who were not matched were excluded.

### IVF treatment

Clomiphene citrate 50 mg per day was administered in conjunction with human menopausal gonadotropin 75 IU per day starting on menstrual cycle day 2 or 3. Patients in the GH group were co-treated with human recombinant GH (Jintropin, JenSci, China) 3 IU per day during COS. When the leading follicle reached a diameter of 18 mm or greater, ovulation triggering was performed with human chorionic gonadotropin (hCG) 10,000 IU, and the number of follicles ≥14 mm was recorded. Oocytes were retrieved 34–36 h later through follicular flushing.

### FF collection and metabolite extraction

To avoid blood contamination, FF from only the follicle with the largest diameter was aspirated first and collected during oocyte retrieval. FF samples were immediately centrifuged at 1000 g, 4 °C for 10 min to remove cellular components. The supernatant was collected, divided into 300 μl aliquots, and stored at − 80 °C until sample preparation. Two hundred fifty microliters aliquots of thawed FF were transferred into 1.5 ml Eppendorf tubes. Subsequently, 20 μl internal standard (2,3,3,3-d4-alanine, 10 mM) was added to each aliquot. To precipitate protein from the follicular sample, 730 μl of cold methanol was added to the follicular aliquots, followed by cooling at − 20 °C for 40 min. Then the supernatant was collected by centrifugation at 1500 g for 20 min and dried in a SpeedVac (LABCONCO, #7810041) at 1000 g for 8 hr. The extracted metabolites were stored at − 80 °C freezer prior to derivatization.

### Methyl chloroformate (MCF) derivatization and gas chromatography-mass spectrometry (GC-MS)

Dried follicular extracts were resuspended in 200 μl of sodium hydroxide (1 M), and MCF derivatization was added to reduce the boiling points of compounds for GC-MS analysis, according to the methodology published by Smart et al. [31]. All follicular samples were analyzed in a single randomized order, and volatile compounds were separated by An Agilent 7890B GC system using a ZB-1701 GC capillary column (30 m × 250 μm id × 0.15 μm with a 5 m guard column, Phenomenex) and analyzed m/z of ions by an Agilent 5977As mass spectrometer (Agilent, California, USA) with electron impact ionization via electron emission at 70 eV. The GC temperature ramps and MS settings were operated according to the protocol published in Han et al. [32].

### Data extraction and normalization

The GC peaks were first deconvoluted by Automated Mass Spectral Deconvolution & Identification System software (AMIDS). The compounds were identified by Ion fragmentation patterns and GC retention time to our in-house mass spectral library built by chemical standards. The MassOmics XCMS R-based software was implemented to extrapolate the relative concentration of the metabolites through the peak height of the most abundant fragmented ion mass [33]. To achieve the reproducibility robustness along with minimizing sample preparation and instrumental variabilities, the relative concentration of the identified metabolites were normalized in the sequence of internal standards (D4-alanine, D5-phenylalanine, or D2-tyrosine), batch correction by quality control of pooled samples, and total ion concentration (TIC) of the follicular metabolome. Lastly, negative blanks were used to eliminate carryover contaminations.

### Statistical analysis

Categorical data were expressed as frequency or percentage, and statistical comparison between groups was carried out using Pearson’s chi-squared test or Fisher’s exact test. The Shapiro–Wilk test was used to test the normal distribution of continuous variables, which were expressed as mean ± standard deviation if normally distributed and as median (25th percentile, 75th percentile) if not normally distributed. Statistical comparison was performed using the Student t-test or Mann–Whitney U test for continuous variables, where appropriate. Prior to statistical analysis of the FF metabolome, metabolite concentration was transformed by log_2_ scale and Pareto scaling to establish Gaussian distribution for this dataset. Partial least squares discriminant analysis (PLS-DA) and model validation were performed by MetaboAnalyst 5.0 (https://www.metaboanalyst.ca/). Since clinical characteristics associated with IVF outcomes were matched between the GH and control groups, Student t-test and false discovery rate were implemented to calculate the significance of FF metabolites between two groups using R software. Only both two-tailed *p-*values and *q*-values less than 0.05 and 0.1 respectively were considered statistically significant. The areas under the receiver operating characteristic (ROC) curve were performed using pROC R package [34]. The forest plot displaying the Pearson correlation between the number of oocytes retrieved and the levels of differential metabolites was illustrated using the ggplot2 R package [35]. The Sankey diagram, which links differential metabolites into their KEGG metabolic pathways, was performed by the Online website https://www.omicstudio.cn/. The metabolic network was illustrated based on the KEGG global metabolism map using MetaboAbalyst 5.0.

## Results

### Clinical characteristics of participants

**Fig. 1:**
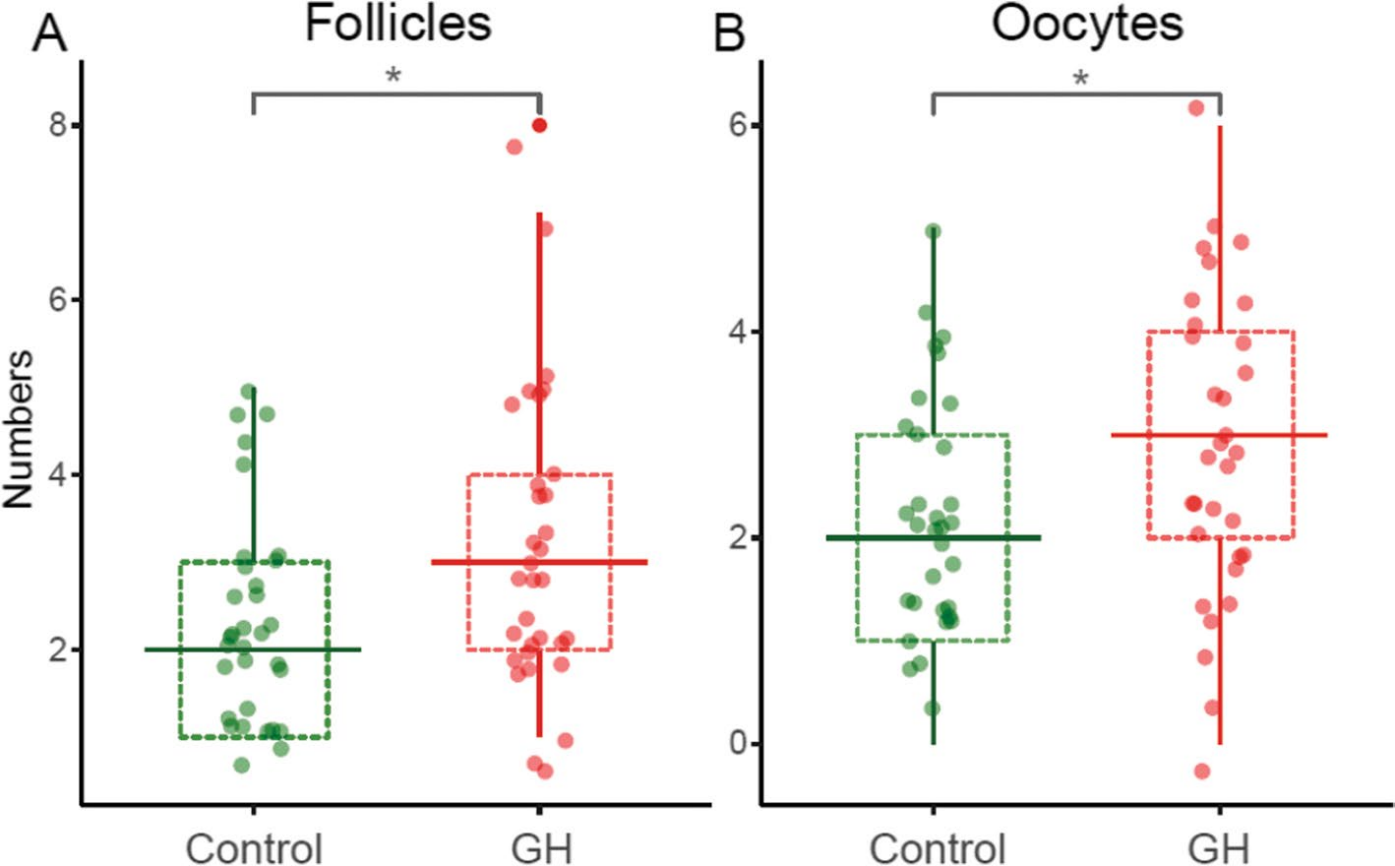
The numbers of follicles ≥14 mm on the day of hCG (**A**) and oocytes retrieved (**B**)

A total of 64 DOR patients, comprising 32 in the GH group and 32 in the control group, were included in this study. The clinical characteristics of the participants are presented in Table 1. The baseline characteristics were comparable between the GH and control groups, including age, BMI, duration of infertility, previous conception, previous IVF attempt, AMH, AFC, in addition to the levels of basal FSH, luteinizing hormone (LH) and estradiol (E2). There was no significant difference in the days of stimulation, FSH dose, or LH dose between both groups. It was noteworthy that the number of follicles above 14 mm on the day of hCG was significantly higher in the GH group compared to the control group (3 vs 2, *p* = 0.03; Fig. 1A). More importantly, this difference was also found for the number of oocytes retrieved between the GH and control groups (3 vs 2, *p* = 0.04; Fig. 1B). Moreover, there were trends toward higher E2 levels on the day of hCG (1380.8 ± 638.2 vs 1160.3 ± 582.6, *p* = 0.15) and normal fertilization rate (82.4% vs 69.1%, *p* = 0.05) in the GH group compared with the control group.

**Table 1:** Clinical characteristics of participants

	Control group (*n* = 32)	GH group (*n* = 32)	*P*-value
Age (years)	34 (31, 38)	33 (32, 37)	0.96^a^
BMI (kg/m^2^)	20.9 (19.5, 23.3)	20.7 (19.9, 21.8)	0.37^a^
Duration of infertility (years)	2 (1.0, 5.3)	4.5 (2.0, 7.3)	0.17^a^
Previous conception	62.5% (20/32)	40.6% (13/32)	0.13^b^
Previous IVF attempt	0 (0, 1)	1 (0, 1)	0.16^a^
AMH (ng/mL)	1.1 (0.9, 1.4)	1.2 (0.9, 1.8)	0.32^a^
AFC	3.5 (2.8, 5.0)	4.0 (3.0, 5.0)	0.39^a^
Basal FSH (IU/L)	9.2 ± 2.7	9.4 ± 2.7	0.80^c^
Basal LH (IU/L)	4.9 (3.7, 5.7)	5.4 (4.2, 5.7)	0.36^a^
Basal E2 (pg/mL)	38.8 (30.7, 63.2)	43.2 (29.8, 59.5)	0.65^a^
Days of stimulation	7 (6.0, 7.3)	7 (5.8, 8.0)	0.96^a^
FSH dose (IU)	637.5 (450.0, 1050.0)	675.0 (450.0, 1050.0)	0.64^a^
LH dose (IU)	450 (150.0, 618.8)	450 (300.0, 618.8)	0.83^a^
E2 on the day of hCG (pg/mL)	1160.3 ± 582.6	1380.8 ± 638.2	0.15^c^
Follicles ≥14 mm on day of hCG	2 (1, 3)	3 (2, 4)	0.03^a^
No. of oocytes retrieved	2 (1, 3)	3 (2, 4)	0.04^a^
Normal fertilization rate	69.1% (47/68)	82.4% (75/91)	0.05^b^
No. of Day 3 available embryo	0 (0, 1)	0 (0, 2)	0.56^a^

Normal fertilization rate: the percentage of 2PN zygotes to oocytes retrieved

Day 3 available embryo: an embryo with ≥5 blastomeres and ≤ 30% fragmentation in 68 ± 1 h after insemination

*Abbreviations*: *BMI* Body mass index, *IVF* In vitro fertilization, *AMH* Anti-Müllerian hormone, *AFC* Antral follicle count, *FSH* Follicle stimulating hormone, *LH* Luteinizing hormone, *E2* Estradiol, *hCG* Human chorionic gonadotropin, *No.* Number

^a^Mann–Whitney U test. The data were expressed as the median (25th percentile, 75th percentile)

^b^Pearson’s chi-squared test. The data were expressed as percentage (numerator/denominator)

^c^Student t-test. The data were expressed as the mean ± SD

### The difference of FF metabolite profiles between the GH and control groups

**Fig. 2:**
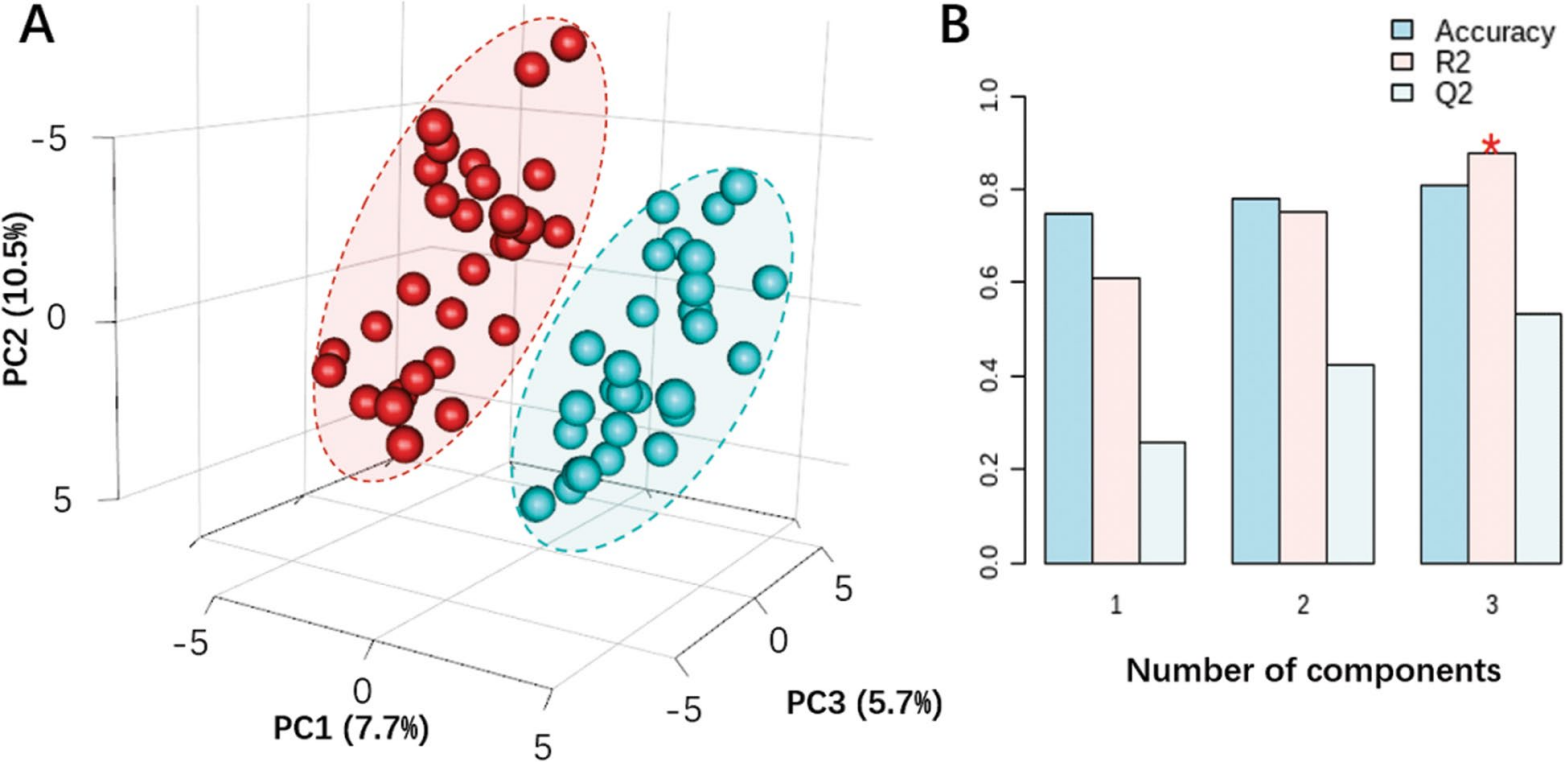
PLS-DA of the FF metabolome between the GH and control groups. The right bar graphs evaluate the prediction model performance via leave-one-out validation. R2 indicates how well the model represents the data. Q2 indicates how reproducible is the PLS-DA model. *PLS-DA* partial least squares discriminant analysis, *FF* follicular fluid, *GH* growth hormone

**Fig. 3:**
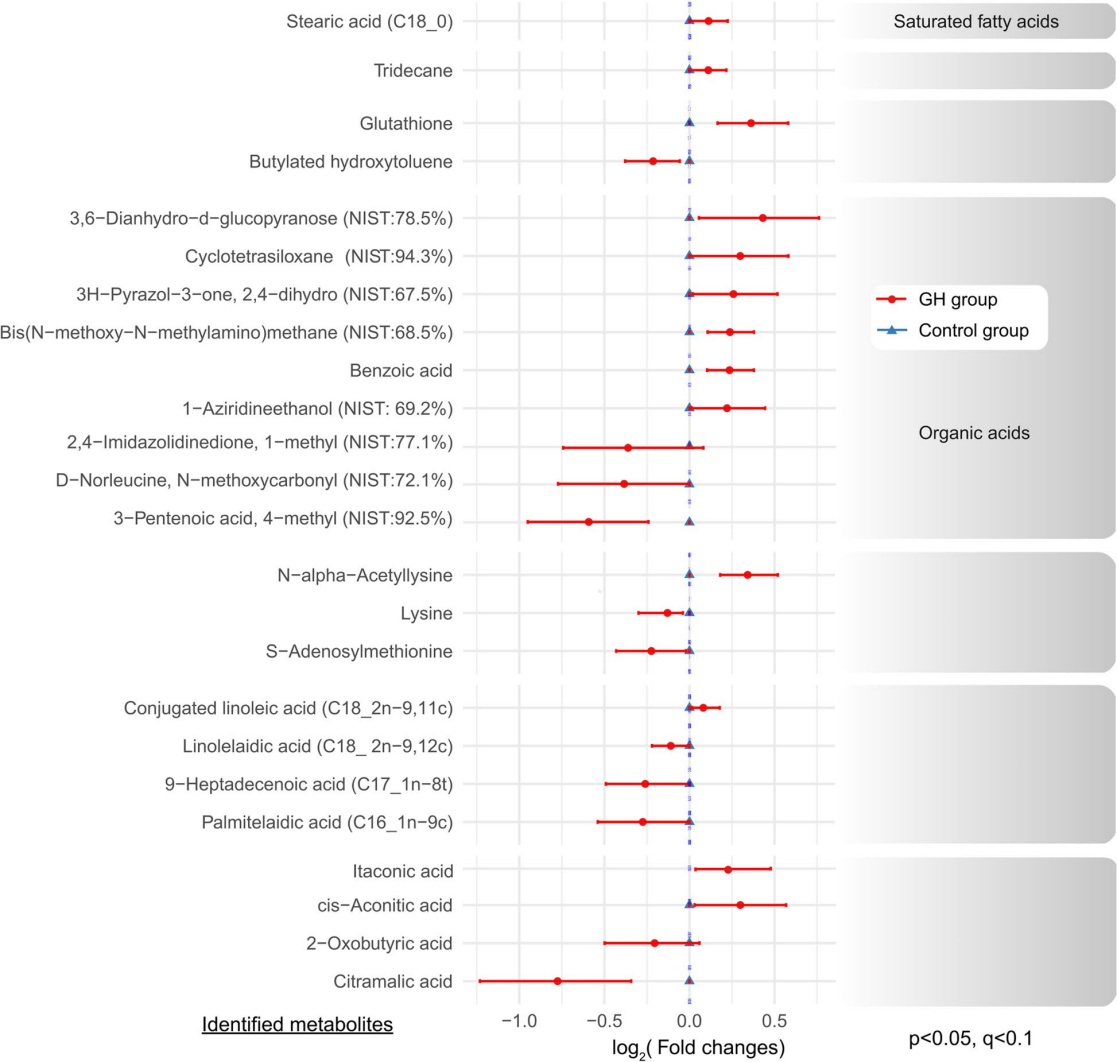
The differences in the FF metabolome between the GH and control groups. The relative concentrations of FF metabolites are illustrated via a log_2_ scale. Positive values represent higher metabolite levels in the dividend group (GH group) than the divisor group (control group), whereas negative values represent lower metabolite levels in the dividend group than the divisor group. Only the metabolites with a *p*-value less than 0.05 (Student t-test) and a *q*-value less than 0.01 (false discovery rate) are displayed. *FF* follicular fluid, *GH* growth hormone, *TCA* tricarboxylic acid

Based on all 134 metabolites identified from FF, the PLS-DA displayed a distinct separation between the GH and control groups via principal components PC1, PC2 and PC3, representing 7.7, 10.5, and 5.7% of the variance respectively (Fig. 2). The leave-one-out validation model showed the best PLS-DA performance using three accumulative PC (Accuracy = 0.83, R2 = 0.88, Q2 = 0.54). Among identified metabolites, 24 of them appeared to be significantly different in concentration between two groups with *p*-value and *q*-value less than 0.05 and 0.1, respectively (Fig. 3 and Table S1). In FF from patients with GH administration, the levels of itaconic acid, glutathione, cis-aconitic acid, N-alpha-acetyllysine, stearic acid, tridecane, and the majority of organic acids were significantly elevated, while those of S-adenosylmethionine (SAM), 2-oxobutyric acid, citramalic acid, and butylated hydroxytoluene were significantly reduced compared with controls. Furthermore, most of the unsaturated-chain fatty acids displayed reduced levels in the GH group, including linolelaidic acid, 9-heptadecenoic acid, and palmitelaidic acid.

### The correlation between the number of oocytes retrieved and the levels of FF metabolites

**Fig. 4:**
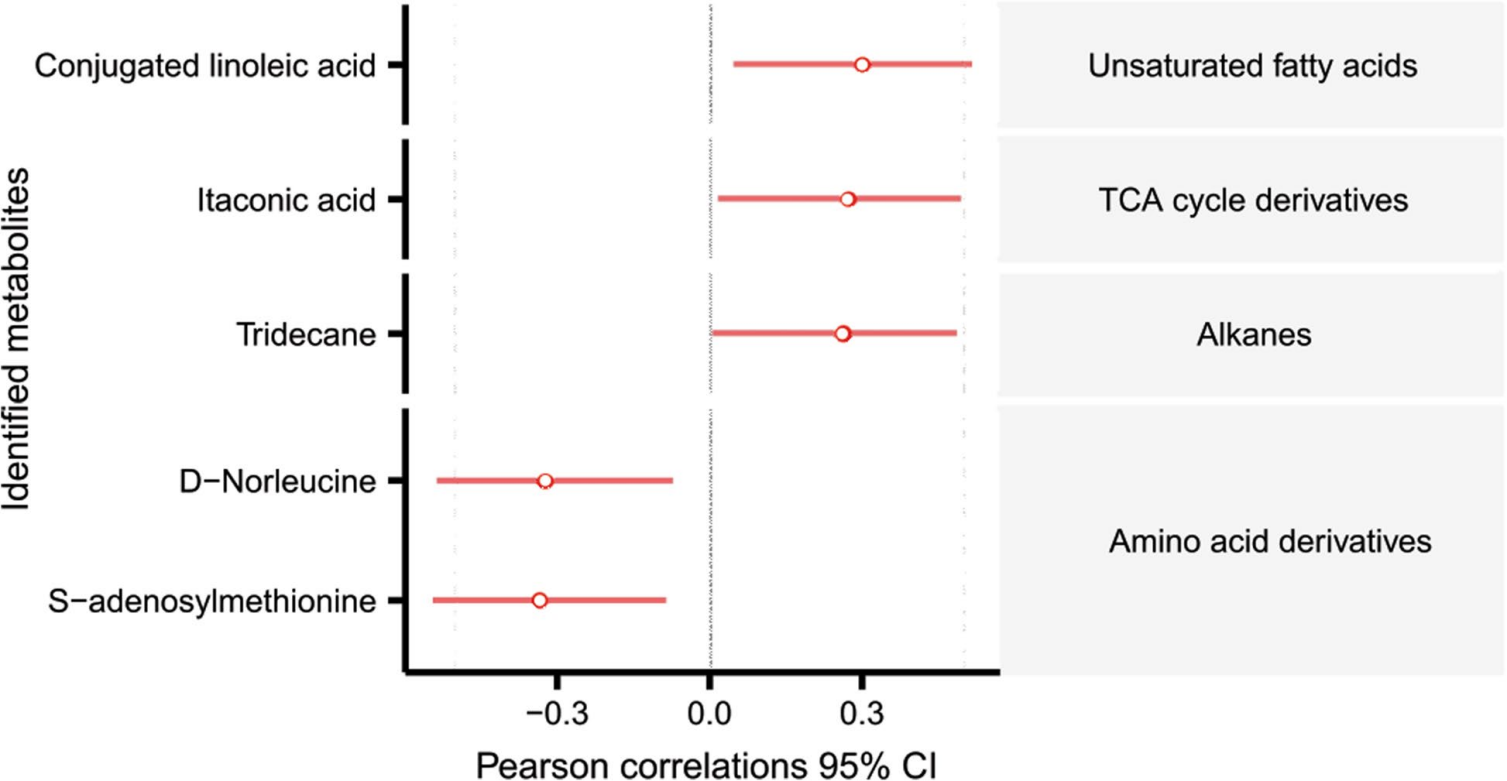
The correlation between the number of oocytes retrieved and the levels of differential metabolites. Statistical analyses were conducted using Pearson correlation. *TCA* tricarboxylic acid, *CI* confidence interval

The number of oocytes retrieved is positively correlated with the levels of conjugated linoleic acid, itaconic acid, and tridecane, while negatively correlated with the levels of D − norleucine and SAM (Fig. 4).

### The metabolic pathway enrichment analysis of differential metabolites

**Fig. 5:**
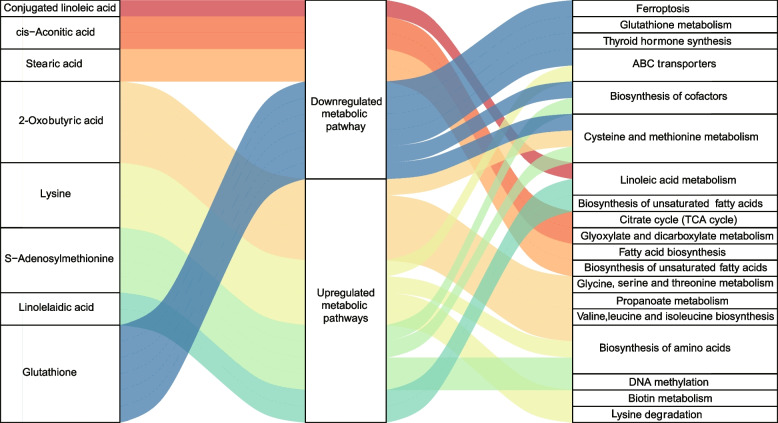
A Sankey diagram displays how differential metabolites participated in upregulated or downregulated KEGG metabolic pathways. *ABC* ATP-binding cassette, *TCA* tricarboxylic acid

**Fig. 6:**
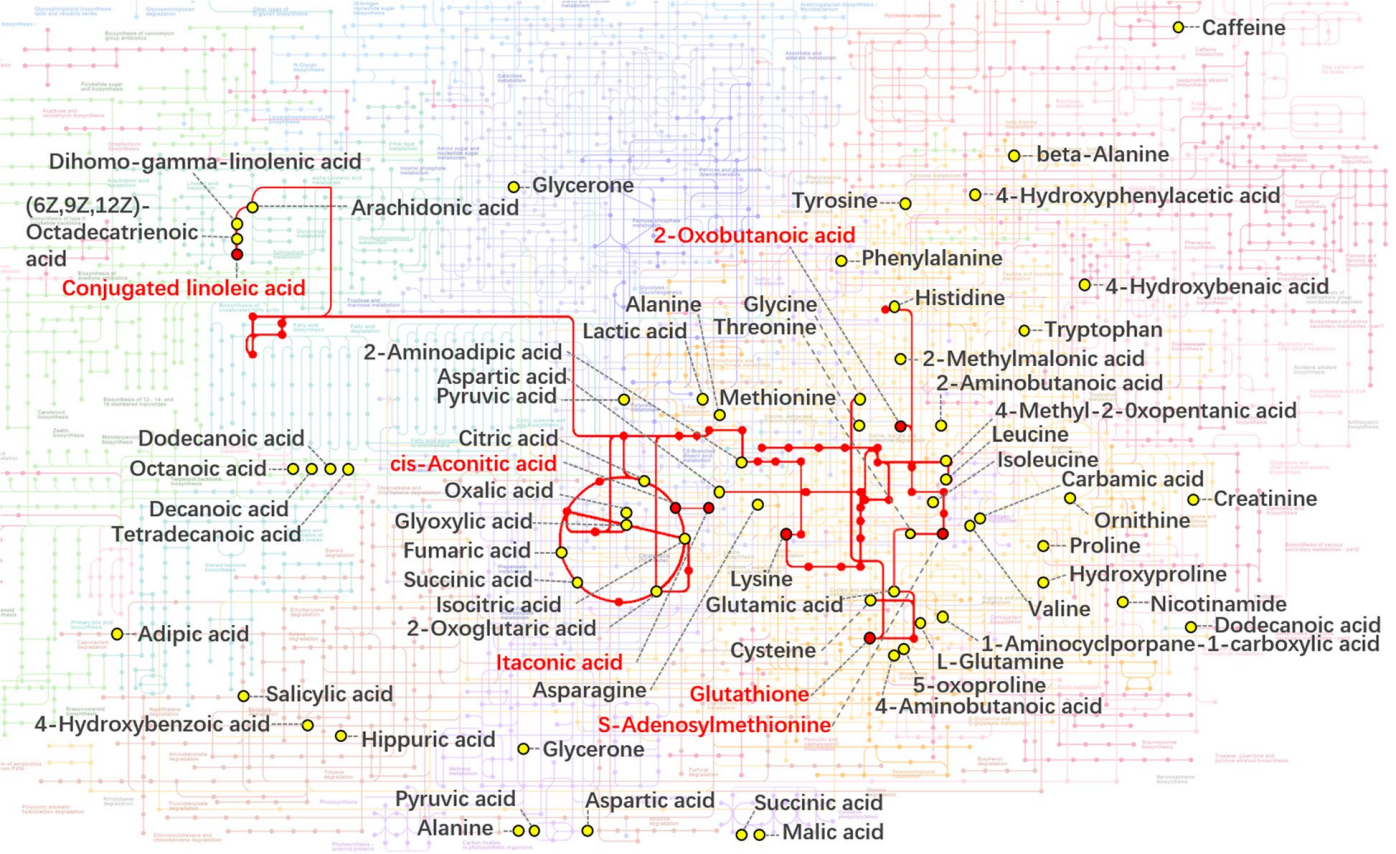
The KEGG metabolic network in which FF metabolites of DOR patients were involved. The metabolic network was based on KEGG global metabolic pathways. The red dots and red letters are the metabolites that were statistically significant between the GH and control groups. The yellow dots and black letters are identified metabolites that displayed no significance. The red lines are metabolic pathways that connect differential metabolites with the shortest distance. *FF* follicular fluid, *DOR* diminished ovarian reserve, *GH* growth hormone

As shown in Fig. 5, these differential metabolites were mapped into their corresponding metabolic pathways in order to investigate the potential biological processes altered by GH. FF metabolites, including conjugated linoleic acid, cis-aconitic acid, stearic acid and glutathione, were involved in various downregulated center carbon metabolism. Especially, glutathione was branched into the most diverse metabolic pathways, namely ferroptosis, glutathione metabolism, thyroid hormone synthesis, ATP-binding cassette (ABC) transporters, biosynthesis of cofactors, as well as cysteine and methionine metabolism. And the other differential metabolites, 2-oxobutyric acid, lysine, SAM, and linolelaidic acid were involved in the upregulation of amino acid or fatty acid metabolism. In addition, SAM is also linked to DNA methylation. The significant metabolic pathways were connected to their common metabolites and constructed in silico into a metabolic network using KEGG global metabolic framework, as displayed in Fig. 6. The metabolic network demonstrated that some differential metabolites could be linked with the shortest distance by the following metabolic pathways: glutathione metabolism, cysteine and methionine metabolism, linoleic acid metabolism, biosynthesis of unsaturated fatty acid, tricarboxylic acid (TCA) cycle, glyoxylate metabolism, and lysine degradation.

## Discussion

This research was the first to employ metabolomics approaches to analyze the effect of GH administration on the FF metabolome in DOR patients undergoing IVF. Our results showed that the co-treatment with GH during COS is involved in altering FF metabolite profiles. Twenty-four differential metabolites were identified between the GH and control groups. Among them, the concentrations of antioxidant metabolites itaconic acid and glutathione were increased by GH administration, while SAM concentration was reduced. In addition, the number of oocytes retrieved increased with GH administration and was correlated with the levels of five differential metabolites, including itaconic acid and SAM. These findings may reveal the potential therapeutic mechanism of GH in improving oocyte development of DOR patients.

All essential intermediates of the TCA cycle were detected in FF. Interestingly, a significantly higher level of itaconic acid was observed in the GH group. Itaconic acid and itaconate generate from the decarboxylation of cis-aconitate, a TCA cycle intermediate. The derivative of itaconic acid, four-octyl itaconate, has been demonstrated to attenuate H_2_O_2_-induced reactive oxygen species (ROS) production, lipid peroxidation and DNA damage, in addition to neuronal cell death and apoptosis [36]. Another study found that itaconate slowed down TCA cycle metabolism and reduced ROS levels to improve brain function by inhibiting succinate dehydrogenase [37]. Oxidative stress due to ROS accumulation in the follicular microenvironment plays a critical role in ovarian aging or DOR development [38–40]. Furthermore, reduced ovarian response to COS is also associated with increased oxidative stress in the follicular microenvironment [41]. Antioxidants, such as coenzyme Q10 and melatonin, improve ovarian response and embryo quality in DOR patients undergoing IVF [42, 43]. Recently, GH was reported to decrease ROS levels in granulosa cells and increase the number of high-quality embryos in patients with poor ovarian response, but the underlying mechanism is unclear [14]. In the present study, the level of itaconic acid was positively correlated with the number of oocytes retrieved. These findings suggest that GH treatment improves ovarian response probably by reducing ROS generation through elevating the levels of itaconic acid in the follicular microenvironment.

The glutathione levels in the GH group were also higher than those in the control group. Likewise, GH treatment led to increased glutathione levels in rat ovary tissues [44]. Glutathione is a powerful antioxidant removing ROS and protects oocytes against oxidative damage and exhibits a beneficial effect on the quality of ovine and bovine oocytes [45–47]. Most importantly, higher glutathione levels in granulosa cells and FF have been found to be associated with increased fertilization potential of oocytes in IVF patients [48, 49]. Moreover, the KEGG metabolic pathway analysis revealed a downregulation of ferroptosis and glutathione catabolism, the latter of which might be owing to the suppression of glutathione degradation enzyme by GH administration [50]. Ferroptosis, an iron-dependent cell death, can be initiated by glutathione depletion [51]. Nowadays, there is growing evidence that ferroptosis may also be a key driver of pathology in age-related diseases [52]. Although little has been reported on the direct association between shortlisted metabolic pathways (glutathione metabolism, ferroptosis, and fatty acid metabolism; Fig. 5) and patients with DOR, Liang et al. demonstrated that oxylipins metabolism was significantly altered in FF of DOR patients [28]. Oxylipins are a class of lipid metabolites that derive from the oxidation of unsaturated fatty acids, and their production can be affected by ROS and fatty acid levels, which are related to our shortlisted metabolic pathways. Taken together, GH may rejuvenate the oocytes of DOR patients by raising glutathione levels and then attenuating oxidative stress in the follicular microenvironment.

Last but not least, there was a significant decrease of SAM in the GH group compared to the control group. This finding is supported by the fact that daily injections of GH inhibited the activity and mRNA transcription of SAM synthetase [53]. Besides a major methyl donor for the methylation of DNA, RNA and histone, SAM is one of the glutathione precursors [54], and our KEGG metabolic network also reveals the connection between them. Recently it was proved that the enhanced biosynthesis of SAM in the germline of Drosophila ovaries led to aging-related defects in oogenesis [55]. Although it has not been evaluated in human ovaries, the SAM biosynthesis is regarded as evolutionarily conserved. In addition, a variety of evidence suggested a strong association between methylation and cell aging [56]. Compared to women with normal ovarian reserve, a distinctive DNA methylation profile was found in mural granulosa cells of DOR patients [57]. In our study, the SAM levels in FF were negatively correlated with the number of oocytes retrieved. Therefore, GH administration may improve ovarian response by reducing SAM biosynthesis in follicles. In concert with the decreased SAM levels induced by GH in this study, GH was reported to result in age- and sex-dependent DNA hypomethylation [58]. Given these findings, it is necessary to notice the possibility of aberrant genomic imprinting, which gives rise to the onset of imprinting disorders in the offspring.

## Conclusions

The findings in this study suggest that the co-treatment with GH during COS alters FF metabolite profiles and in turn increases the number of oocytes retrieved in DOR patients. The novel data reported here show the elevating effect of GH on the antioxidant metabolites itaconic acid and glutathione. In addition, the levels of SAM, a regulator of genomic imprinting, were downregulated by GH treatment. In future work, the antioxidant capability of itaconic acid/glutathione and SAM synthetase activity regulated by GH should be validated. Combined with previous findings, GH has been proved to improve IVF outcomes, but we should be aware of the potential risk of imprinting disturbances. And large multi-center cohort studies are warranted to evaluate this possible harmful effect of GH on offsprings.

## Supplementary Information

**Additional file 1: Table S1.** The area under the receiver operating characteristic curve (AUC), *p*-value, *q*-value and peak intensity of identified metabolites.

## Data Availability

The datasets used and/or analyzed during the current study are available from the corresponding author upon reasonable request.

## Abbreviations

ABC: ATP-binding cassette
AFC: Antral follicle count
AMH: Anti-Müllerian hormone
AMIDS: Automated Mass Spectral Deconvolution & Identification System software
AUC: Area under the receiver operating characteristic curve
BMI: Body mass index
COS: Controlled ovarian stimulation
DHEA: Dehydroepiandrosterone
DOR: Diminished ovarian reserve
E2: Estradiol
FF: Follicular fluid
FSH: Follicle-stimulating hormone
GC-MS: Gas chromatography-mass spectrometry
GH: Growth hormone
GHR: Growth hormone receptor
hCG: Human chorionic gonadotropin
IVF: In vitro fertilization
LH: Luteinizing hormone
MCF: Methyl chloroformate
ROC: Receiver operating characteristic
PLS-DA: Partial least squares discriminant analysis
POR: Poor ovarian reserve
PC: Principal components
ROS: Reactive oxygen species
SAM: S-adenosylmethionin
TCA: Tricarboxylic acid
TIC: Total ion concentration
