# Supplementary material for: The effect of growth hormone on the metabolome of follicular fluid in patients with diminished ovarian reserve
Source: Reprod Biol Endocrinol. 2023 Feb 27;21:21. doi: 10.1186/s12958-023-01073-x (PMC9969693; doi:10.1186/s12958-023-01073-x)
Supplement: Supplementary file 1 — Additional file 1: Table S1. The area under the receiver operating characteristic curve (AUC), p-value, q-value and peak intensity of identified metabolites. [file 12958_2023_1073_MOESM1_ESM.docx]

**Table S1**. The area under the receiver operating characteristic curve (AUC), *p*-value, *q*-value and peak intensity of identified metabolites

| **Name of metabolites** | **AUC** | ***p*-value** | ***q*-value** | **peak intensity** | |
| --- | --- | --- | --- | --- | --- |
|  |  |  |  | **control group** | **GH group** |
| (1-Methyl-2,6-dioxocyclohexyl) acetic acid (NIST:52.8%) | 0.63 | 0.03 | 0.11 | 4816.83 ± 3491.07 | 2987.02 ± 1497.47 |
| 10,13-dimethyltetradecanoic acid (C17_0) | 0.70 | 0.03 | 0.11 | 2436531.44 ± 159577.32 | 2550249.44 ± 212195.97 |
| 11,14,17-Eicosatrienoic acid (C20_3n-3,6,9c) | 0.60 | 0.32 | 0.38 | 493371.66 ± 124434.60 | 531374.17 ± 124419.94 |
| 11,14-Eicosadienoic (C20_2n-6,9c) | 0.49 | 0.84 | 0.59 | 30957.99 ± 6555.84 | 30596.58 ± 6174.30 |
| 16.7644 min N-(Carboxymethyl)-L-alanine | 0.52 | 1.00 | 0.63 | 4297.04 ± 2076.96 | 4203.42 ± 1726.68 |
| 1-Aminocyclopropane-1-carboxylic acid | 0.52 | 0.54 | 0.47 | 1863.87 ± 405.23 | 2053.98 ± 791.70 |
| 1-Aziridineethanol (69.3%) | 0.66 | 0.02 | 0.09 | 7752.15 ± 1633.47 | 9033.26 ± 2341.32 |
| 1-Oxa-3,4-diazacyclopentadiene (NIST:65.7%) | 0.64 | 0.09 | 0.20 | 11724.62 ± 2774.45 | 13314.47 ± 3651.47 |
| 2,3-Butanediol | 0.52 | 0.63 | 0.51 | 26356.61 ± 9588.72 | 28593.84 ± 12903.96 |
| 2,4-Di-tert-butylphenol | 0.54 | 0.49 | 0.47 | 42584.22 ± 6308.30 | 41441.21 ± 5188.74 |
| 2,4-Imidazolidinedione, 1-methyl (NIST:77.0%) | 0.65 | 0.02 | 0.09 | 5028.81 ± 2066.51 | 3916.27 ± 2012.62 |
| 2-Aminoadipic acid | 0.68 | 0.04 | 0.11 | 3441.77 ± 1546.22 | 2779.62 ± 1073.88 |
| 2-Aminobutyric acid | 0.54 | 0.61 | 0.50 | 318590.59 ± 102928.28 | 304220.82 ± 97890.41 |
| 2-Hydroxybutyric acid | 0.58 | 0.56 | 0.48 | 118931.75 ± 38818.71 | 113235.16 ± 42223.40 |
| 2-Hydroxyglutaramic acid | 0.61 | 0.11 | 0.20 | 13600.84 ± 3931.92 | 12055.59 ± 2917.33 |
| 2-Hydroxyisobutyric acid | 0.55 | 0.50 | 0.47 | 1036.59 ± 319.84 | 989.85 ± 348.89 |
| 2-Methyloctadecanoic acid | 0.57 | 0.30 | 0.37 | 2940.39 ± 1113.00 | 2609.45 ± 804.16 |
| 2-Oxobutyric acid | 0.71 | 0.01 | 0.07 | 8121.34 ± 1253.13 | 7046.72 ± 2247.67 |
| 2-Oxoglutaric acid | 0.63 | 0.11 | 0.20 | 90574.69 ± 11448.85 | 85417.83 ± 15139.61 |
| 2-Oxomalonic acid, methylhydrazone (NIST:62%) | 0.78 | 0.00 | 0.01 | 79956.68 ± 24561.23 | 105788.48 ± 25530.32 |
| 2-Oxovaleric acid | 0.49 | 0.67 | 0.53 | 88103.14 ± 36031.56 | 105725.93 ± 108704.42 |
| 2-t-Butyl-5-methyl-oxazole-3-carboxylic acid, methyl ester (NIST:49.9%) | 0.67 | 0.03 | 0.10 | 4132.67 ± 946.72 | 4852.84 ± 1329.03 |
| 3,6-Dianhydro-D-glucopyranose (NIST:78.6%) | 0.68 | 0.01 | 0.07 | 1021.08 ± 360.85 | 1377.07 ± 705.64 |
| 3H-Pyrazol-3-one, 2,4-dihydro (NIST:67.6%) | 0.69 | 0.01 | 0.07 | 1136.16 ± 206.31 | 1359.16 ± 384.28 |
| 3-Hydroxydecanoic acid | 0.57 | 0.49 | 0.47 | 1731.25 ± 651.72 | 1608.62 ± 473.82 |
| 3-Methyl-2-oxopentanoic acid | 0.52 | 0.93 | 0.60 | 147261.93 ± 33685.62 | 145047.97 ± 26025.91 |
| 3-Pentenoic acid, 4-methyl (NIST:92.6%) | 0.68 | 0.01 | 0.08 | 17398.08 ± 9990.72 | 11541.03 ± 7197.97 |
| 4-Aminobutyric acid (GABA) | 0.49 | 0.36 | 0.39 | 24713.33 ± 1494.65 | 24273.09 ± 2777.82 |
| 4-Hydroxyphenylacetic acid | 0.59 | 0.15 | 0.25 | 3516.86 ± 1418.09 | 4641.06 ± 3069.49 |
| D_4-Methyl-2-oxopentanoic acid | 0.57 | 0.10 | 0.20 | 1816.78 ± 1226.02 | 1386.03 ± 653.87 |
| 4-Methyl-2-oxopentanoic acid | 0.56 | 0.47 | 0.46 | 183384.23 ± 40962.86 | 174609.77 ± 30665.75 |
| 5-Oxotetrahydrofuran-2-carboxylic acid | 0.57 | 0.34 | 0.39 | 4246.19 ± 731.82 | 4415.26 ± 671.65 |
| 9-Amino-1-phenyl-3,6-diazahomoadamantane (NIST:49.0%) | 0.60 | 0.08 | 0.19 | 3033.85 ± 1454.32 | 2465.61 ± 1352.99 |
| 9-Heptadecenoic acid (C17_1n-8t) | 0.68 | 0.01 | 0.08 | 5421.25 ± 1608.37 | 4527.72 ± 593.43 |
| Adipic acid | 0.59 | 0.32 | 0.38 | 40555.57 ± 11092.8 | 38309.47 ± 13163.23 |
| Adrenic acid (C22_4n-6,9,12,15c) | 0.63 | 0.05 | 0.16 | 20113.08 ± 7124.46 | 16784.35 ± 5872.29 |
| Alanine | 0.50 | 0.87 | 0.60 | 3434472.67 ± 475070.23 | 3415271.88 ± 472736.27 |
| Arachidic acid (C20_0) | 0.46 | 0.35 | 0.39 | 4718.95 ± 1138.34 | 5161.23 ± 1666.37 |
| Arachidonic acid (C20_4n-6,9,12,15c) | 0.60 | 0.32 | 0.38 | 493371.66 ± 124434.6 | 531374.17 ± 124419.94 |
| Asparagine | 0.56 | 0.33 | 0.38 | 193068.29 ± 48592.85 | 206864.1 ± 60120.97 |
| Aspartic acid | 0.58 | 0.52 | 0.47 | 109767.22 ± 78562.94 | 127162.75 ± 124432.69 |
| Azelaic acid | 0.70 | 0.03 | 0.11 | 691.30 ± 368.77 | 910.51 ± 583.12 |
| Benzoic acid | 0.79 | 0.00 | 0.00 | 5909.2 ± 570.54 | 6957.58 ± 1093.01 |
| beta-Alanine | 0.56 | 0.44 | 0.45 | 9594.56 ± 4789.74 | 8741.42 ± 4891.57 |
| Bis(N-methoxy-N-methylamino) methane (NIST:68.6%) | 0.73 | 0.00 | 0.01 | 27937.29 ± 3663 | 32948.91 ± 6351.18 |
| bishomo-gamma-Linolenic acid (C20_3n-6,9,12c) | 0.50 | 0.81 | 0.58 | 108501.03 ± 37165.19 | 107221.13 ± 38215.65 |
| Butylated hydroxytoluene | 0.75 | 0.00 | 0.03 | 961.03 ± 152.31 | 828.72 ± 175.04 |
| Cabamic acid | 0.60 | 0.26 | 0.35 | 2644.67 ± 490.96 | 2764.12 ± 401.91 |
| Caffeine | 0.58 | 0.57 | 0.48 | 6482.13 ± 22359.05 | 2246.96 ± 5684.29 |
| cis-4-Hydroxyproline | 0.44 | 0.47 | 0.46 | 67897.93 ± 59517.43 | 53613.58 ± 30202.92 |
| cis-Aconitic acid | 0.69 | 0.01 | 0.07 | 8324.34 ± 2207.53 | 10242.89 ± 2933.10 |
| Citraconic acid | 0.48 | 0.49 | 0.47 | 881.83 ± 304.88 | 1026.29 ± 788.14 |
| Citramalic acid | 0.69 | 0.02 | 0.09 | 2945.22 ± 2915.72 | 1720.95 ± 2142.70 |
| Citric acid | 0.60 | 0.10 | 0.20 | 245411.97 ± 55948.77 | 271485.62 ± 64512.16 |
| Citric acid secondary peak | 0.49 | 0.72 | 0.55 | 2332.24 ± 772.21 | 2326.40 ± 906.39 |
| Conjugated linoleic acid (C18_2n-9,11c) | 0.67 | 0.03 | 0.10 | 1296068.90 ± 108450.44 | 1371314.98 ± 130077.27 |
| Creatinine | 0.50 | 0.90 | 0.60 | 11191.31 ± 4320.92 | 11385.70 ± 4724.82 |
| cyclopenta[b]quinoxaline-1,2,3-trione (NIST:55.6%) | 0.71 | 0.02 | 0.09 | 2203.26 ± 951.98 | 2568.55 ± 779.44 |
| Cyclotetrasiloxane (NIST:94.4%) | 0.75 | 0.00 | 0.03 | 5472.44 ± 1278.02 | 6731.49 ± 2112.27 |
| Cysteine | 0.53 | 0.51 | 0.47 | 39209.78 ± 22479.73 | 31453.42 ± 9327.00 |
| DBP | 0.63 | 0.09 | 0.20 | 5213.73 ± 1806.99 | 4354.79 ± 820.22 |
| Decanoic acid (C10_0) | 0.52 | 0.59 | 0.49 | 20136.97 ± 6674.34 | 19441.44 ± 7041.42 |
| DHA (C22_6n-3,6,9,12,15,18c) | 0.61 | 0.20 | 0.32 | 208613.51 ± 69727.76 | 229839.72 ± 60454.82 |
| Dimethyl aminomalonic acid | 0.65 | 0.09 | 0.20 | 8247.09 ± 3063.15 | 9336.24 ± 2724.97 |
| Dimethyl fumarate (NIST:93.1%) | 0.66 | 0.07 | 0.18 | 6242.40 ± 1152.79 | 6940.51 ± 1560.95 |
| DL-gamma-methyl-ketoglutaramate | 0.62 | 0.16 | 0.25 | 11110.24 ± 2208.65 | 10286.57 ± 1678.19 |
| D-Norleucine, N-methoxycarbonyl (NIST:72.2%) | 0.68 | 0.01 | 0.07 | 4936.29 ± 1760.35 | 3788.58 ± 1488.12 |
| Dodecane | 0.52 | 0.79 | 0.58 | 13601.12 ± 1237.24 | 13691.40 ± 1307.54 |
| Dodecanoic acid (C12_0) | 0.52 | 0.86 | 0.60 | 53385.84 ± 40211.56 | 47823.35 ± 17335.93 |
| DPA (C22_5n-3,6,9,12,15c) | 0.53 | 0.87 | 0.60 | 33770.00 ± 11448.37 | 34563.07 ± 12111.61 |
| EPA (C20_5n-3,6,9,12,15c) | 0.62 | 0.12 | 0.21 | 26790.54 ± 14451.43 | 32180.39 ± 16894.93 |
| Erucic acid (C22_1n-9c) | 0.51 | 0.91 | 0.60 | 12152.72 ± 3789.65 | 12401.59 ± 4156.87 |
| Fumaric acid | 0.65 | 0.06 | 0.18 | 5850.50 ± 1034.28 | 6474.61 ± 1312.31 |
| gamma-Linolenic acid (C18_3n-6,9,12c) | 0.48 | 0.91 | 0.60 | 27292.71 ± 10050.78 | 28034.86 ± 11184.55 |
| Glutamic acid | 0.62 | 0.10 | 0.20 | 215472.96 ± 72532.12 | 243521.82 ± 62261.46 |
| Glutamine | 0.58 | 0.11 | 0.20 | 52917.73 ± 11863.51 | 47962.67 ± 14421.14 |
| Glutathione | 0.76 | 0.00 | 0.01 | 53515.47 ± 12062.86 | 68782.74 ± 17837.82 |
| Glycine | 0.59 | 0.25 | 0.35 | 1077304.57 ± 228757.72 | 1169428.94 ± 304312.63 |
| Glyoxylic acid | 0.51 | 0.90 | 0.60 | 2306.07 ± 808.11 | 2252.39 ± 693.56 |
| Gondoic acid (C20_1n-9c) | 0.54 | 0.72 | 0.55 | 98417.22 ± 40298.26 | 102707.63 ± 39583.40 |
| Hexanoic acid (C6_0) | 0.54 | 1.00 | 0.63 | 84934.46 ± 63848.73 | 113021.32 ± 185821.67 |
| Hippuric acid | 0.56 | 0.47 | 0.46 | 10291.14 ± 7986.99 | 12691.33 ± 10981.30 |
| Histidine | 0.53 | 0.60 | 0.49 | 116887.58 ± 13924.42 | 115207.66 ± 17630.38 |
| Hydroxybenzoic acid | 0.53 | 0.93 | 0.60 | 942.21 ± 497.28 | 980.21 ± 765.73 |
| Isocitric acid | 0.46 | 0.35 | 0.39 | 589.41 ± 107.30 | 619.03 ± 125.39 |
| Isoleucine | 0.58 | 0.24 | 0.35 | 219422.61 ± 35037.32 | 208694.34 ± 36120.06 |
| Itaconic acid | 0.73 | 0.01 | 0.07 | 2766.17 ± 744.60 | 3151.86 ± 500.08 |
| Lactic acid | 0.55 | 0.42 | 0.45 | 3593947.30 ± 419939.33 | 3506336.46 ± 482151.54 |
| Leucine | 0.63 | 0.07 | 0.18 | 817473.03 ± 111812.15 | 761904.05 ± 129083.84 |
| Linolelaidic acid (C18_ 2n-9,12c) | 0.67 | 0.02 | 0.08 | 39876.48 ± 4874.89 | 36950.18 ± 3737.01 |
| l-Prolylglycine, N-methoxycarbonyl (NIST:85.3%) | 0.62 | 0.06 | 0.18 | 232850.10 ± 75101.96 | 201547.84 ± 78705.67 |
| Lysine | 0.66 | 0.02 | 0.09 | 879484.46 ± 123131.74 | 804075.72 ± 127734.08 |
| Malic acid peak 1 | 0.55 | 0.80 | 0.58 | 6676.10 ± 1836.52 | 6869.72 ± 1873.05 |
| Malic acid peak 2 | 0.55 | 0.78 | 0.58 | 5609.63 ± 1413.46 | 5742.68 ± 1472.98 |
| Malonic acid | 0.60 | 0.13 | 0.23 | 3219.47 ± 517.02 | 3573.76 ± 915.69 |
| Margaric acid (C17_0) | 0.50 | 0.88 | 0.60 | 80471.42 ± 20737.66 | 80798.54 ± 19007.10 |
| Methionine | 0.51 | 0.88 | 0.60 | 65507.95 ± 9672.70 | 65401.57 ± 11392.03 |
| Methylthioacetic acid | 0.59 | 0.53 | 0.47 | 728.67 ± 272.08 | 761.04 ± 223.22 |
| Myristic acid (C14_0) | 0.53 | 0.25 | 0.35 | 152604.70 ± 34193.75 | 142537.94 ± 20233.11 |
| Myristoleic acid (C14_1n-5c) | 0.47 | 0.29 | 0.37 | 30880.24 ± 6512.19 | 29086.80 ± 4035.84 |
| N-(2-Cyanoethyl)-N-(2-hydroxypropyl) nitramine nitrate (ester) (NIST:85.6%) | 0.62 | 0.07 | 0.18 | 14982.45 ± 7394.97 | 12069.27 ± 6289.88 |
| N-Acetylhistidine, methyl ester (NIST:56.2%) | 0.64 | 0.03 | 0.10 | 2418.40 ± 507.55 | 2967.69 ± 1134.19 |
| N-Acetyl-L-lysine-N-methylamide (NIST:53.1%) | 0.70 | 0.01 | 0.08 | 2784.00 ± 565.74 | 3237.01 ± 746.61 |
| N-alpha-Acetyllysine | 0.83 | 0.00 | 0.00 | 3065.96 ± 414.60 | 3886.14 ± 903.31 |
| Nicotinamide | 0.59 | 0.30 | 0.38 | 4280.34 ± 509.27 | 4387.45 ± 380.15 |
| Nonadecanoic acid (C19_0) | 0.57 | 0.28 | 0.37 | 3879.66 ± 878.37 | 4269.24 ± 1259.67 |
| Norleucine | 0.65 | 0.08 | 0.19 | 1789.96 ± 1870.01 | 2549.04 ± 2610.95 |
| Norvaline | 0.64 | 0.11 | 0.20 | 1042.66 ± 967.29 | 1595.43 ± 1975.57 |
| Octanoic acid (C8_0) | 0.56 | 0.97 | 0.62 | 18408.43 ± 16015.81 | 16796.89 ± 7524.94 |
| Oleic acid (C18_1n-9c) | 0.64 | 0.06 | 0.18 | 816293.30 ± 69705.22 | 854731.75 ± 78119.54 |
| Ornithine | 0.59 | 0.25 | 0.35 | 186330.46 ± 92014.78 | 165639.13 ± 47934.64 |
| Oxalic acid | 0.57 | 0.47 | 0.46 | 8160.75 ± 1996.55 | 8924.47 ± 3031.86 |
| Palmitelaidic acid (C16_1n-9c) | 0.67 | 0.01 | 0.07 | 33844.31 ± 9892.84 | 27986.15 ± 5940.67 |
| Palmitoleic acid (C16_1n-7c) | 0.48 | 0.77 | 0.58 | 6826.40 ± 1480.99 | 6979.23 ± 1653.10 |
| Pentadecanoic acid (C15_0) | 0.50 | 0.91 | 0.60 | 24773.89 ± 8524.49 | 24022.52 ± 5729.91 |
| Phenethyl acetate | 0.46 | 0.57 | 0.48 | 3663.82 ± 3446.08 | 4186.71 ± 3615.00 |
| Phenylalanine | 0.55 | 0.39 | 0.41 | 392499.77 ± 49012.19 | 381764.40 ± 62528.46 |
| Phthalic acid, di(2-propylpentyl) ester (NIST:89.5%) | 0.64 | 0.08 | 0.19 | 5109.57 ± 578.70 | 4827.95 ± 708.54 |
| Proline | 0.61 | 0.26 | 0.35 | 3158817.12 ± 635363.65 | 2978607.32 ± 690139.72 |
| Pyroglutamic acid | 0.44 | 0.19 | 0.30 | 175352.58 ± 35820.94 | 163796.09 ± 33714.25 |
| Pyruvic acid | 0.52 | 0.83 | 0.59 | 108192.73 ± 30589.81 | 107574.99 ± 21317.27 |
| S-Adenosylmethionine | 0.67 | 0.01 | 0.07 | 35235.48 ± 7576.31 | 30166.46 ± 6984.36 |
| Salicylic acid | 0.65 | 0.08 | 0.19 | 2062.37 ± 6826.25 | 648.80 ± 488.90 |
| Serine | 0.58 | 0.29 | 0.37 | 25659.03 ± 8418.68 | 23235.76 ± 6407.47 |
| Succinic acid | 0.53 | 0.66 | 0.53 | 31420.37 ± 11351.27 | 32063.20 ± 9807.78 |
| Threonine | 0.47 | 0.53 | 0.47 | 318985.04 ± 117915.81 | 299490.27 ± 102073.40 |
| trans-4-Hydroxyproline | 0.63 | 0.15 | 0.25 | 196500.03 ± 102030.09 | 154149.43 ± 68900.25 |
| trans-Vaccenic acid | 0.59 | 0.22 | 0.34 | 617982.00 ± 58713.21 | 599569.64 ± 53654.44 |
| Tricosane | 0.45 | 0.69 | 0.54 | 1600.20 ± 869.72 | 1847.85 ± 1263.37 |
| Tridecane | 0.73 | 0.00 | 0.03 | 4510.93 ± 369.33 | 4870.53 ± 462.49 |
| Tridecane (C18_0) | 0.69 | 0.02 | 0.09 | 2209663.60 ± 236639.74 | 2388727.93 ± 301486.60 |
| Tryptophan | 0.60 | 0.29 | 0.37 | 558388.36 ± 88243.89 | 535289.39 ± 128276.60 |
| Tyrosine | 0.60 | 0.14 | 0.24 | 338412.28 ± 56428.77 | 316087.71 ± 74788.19 |
| Valine | 0.61 | 0.11 | 0.20 | 2126147.95 ± 261736.90 | 2012781.15 ± 296367.49 |
